# Does neural face processing explain effects of an attachment‐based intervention on maternal sensitivity? A randomized controlled study including pre‐ and postintervention measures

**DOI:** 10.1002/brb3.1972

**Published:** 2021-12-08

**Authors:** Laura Kolijn, Bianca G. van den Bulk, Saskia Euser, Marian J. Bakermans‐Kranenburg, Marinus H. van IJzendoorn, Rens Huffmeijer

**Affiliations:** ^1^ Department of Clinical Child and Family Studies, and Amsterdam Public Health Vrije Universiteit Amsterdam Van der Boechorststraat 7 Amsterdam North Holland 1081 BT The Netherlands; ^2^ Leiden Consortium on Individual Development Leiden University Leiden The Netherlands; ^3^ Leiden Institute for Brain and Cognition Leiden University Leiden The Netherlands; ^4^ Erasmus University Rotterdam The Netherlands; ^5^ Department of Psychology, Education and Child Studies Erasmus University Rotterdam Rotterdam The Netherlands; ^6^ Institute of Education and Child Studies Leiden University Leiden The Netherlands

**Keywords:** ERP, maternal sensitivity, mediation, N170, parenting behavior, VIPP‐SD

## Abstract

**Background:**

Although there is a large body of literature highlighting the behavioral effects of parenting interventions, studies on the neurocognitive mechanisms involved in such intervention effects remain scarce.

**Purpose:**

The aim of the current study was to test whether changes in neural face processing (as reflected in N170 amplitudes) would act as a mediator in the association between the Video‐feedback Intervention to promote Positive Parenting and Sensitive Discipline (VIPP‐SD) and maternal sensitivity.

**Methods:**

A total of 66 mothers of whom a random 33% received the VIPP‐SD and the others a “dummy” intervention participated in pre‐ and postintervention assessments. We recorded mothers' electroencephalographic (EEG) activity in response to photographs of children's neutral, happy, and angry facial expressions. Maternal sensitivity was observed while mothers interacted with their offspring in a semi‐structured play situation.

**Results:**

In contrast with our expectations, we did not find evidence for mediation of intervention effects on maternal sensitivity by the N170.

**Conclusion:**

We discuss that parenting support programs may yield different effects on neurocognitive processes depending on the population and provide recommendations for future research. Our study underscores the importance of reporting null findings and preregistering studies in the field of neurocognitive research.

## Graphical abstract

The VIPP‐SD program reduced N170 responses to children's happy and angry expressions, reflecting more efficient face processing. Neural face processing did not mediate intervention effects on maternal behavior. Parenting support programs may yield different effects on neurocognitive processes depending on the population that is examined.

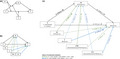

## 1 INTRODUCTION

Parental sensitivity positively affects children's attachment security which in turn contributes to positive child development (Cassidy & Shaver, 2016; Groh et al., 2017). Parenting support programs such as Incredible Years (Gardner et al., 2019; Webster‐Stratton, 2006), Attachment and Biobehavioral Catch‐up (ABC; Dozier et al., 2017), and the Video‐feedback Intervention to promote Positive Parenting and Sensitive Discipline (VIPP‐SD; Juffer et al., 2017) have been found to be effective in enhancing parental sensitivity on a behavioral level. The Consortium on Individual Development (CID; https://individualdevelopment.nl) aims to understand how child characteristics (e.g., genetic, endocrine and neural processes), environmental influences (e.g., home environment, parenting quality), and their interaction contribute to child development. Specifically, the L‐CID branch of the CID tree aims to investigate how parenting influences the development of children's social competence and behavior regulation by enhancing parental sensitivity with the VIPP‐SD program (Juffer et al., 2017). The current study zooms in on processes in parents by investigating whether intervention effects on parenting behavior are only accompanied or also mediated by changes on a neural level. As previously reported (Kolijn et al., 2020), the VIPP‐SD affected mothers' neural processing of emotional child faces. We found smaller N170 amplitudes in the intervention compared to the control group, likely reflecting more efficient information processing after the intervention. In the current study, we test whether this change in N170 amplitudes (partially) explains effects of the VIPP‐SD on maternal sensitivity using mediation analyses.

### 1.1 Parental sensitivity and child development

Parental sensitivity is defined as the ability to perceive, interpret, and respond to children's emotional signals (including facial expressions) and is characterized by prompt, appropriate, and consistent caregiving responses (Ainsworth et al., 1974). It is an important predictor of children's attachment security (e.g., Verhage et al., 2016) and related to positive child outcomes. For example, maternal sensitivity is positively associated with children's cognitive development (Bernier et al., 2010; Malmberg et al., 2016; Merz et al., 2017), social competence (Daniel et al., 2016; Krevans & Gibbs, 1996; Newton et al., 2014), and behavior regulation (Moss et al., 2011; van Zeijl et al., 2006). In addition, there is evidence from both animal and human studies that suggests neurodevelopmental changes may be involved in bringing about these effects (Kok et al., 2015; Rilling & Young, 2014). However, the nature of most of these studies is correlational, limiting the conclusions that can be drawn about causality and the direction of effects.

Attachment‐based interventions, such as the VIPP‐SD, have been found to be effective in enhancing parental sensitivity and attachment security (Juffer et al., 2017). Whereas the original VIPP is suited for parents with infants, the VIPP‐SD (VIPP with an additional focus on Sensitive Discipline) is tailored to parents with children older than 1 year of age, who display more challenging behavior than infants. The foundation of the VIPP‐SD can be found in a combination of two research traditions: Bowlby's attachment theory as a basis for the sensitivity focus, and Patterson's social learning theory for discipline or limit setting (Bowlby, 1982, 1988; Patterson, 1982). The intervention aims to enhance parents' sensitivity to children's (emotional) signals on the one hand (to promote attachment security) and enhance sensitive but firm limit setting on the other hand (to prevent or decrease oppositional child behavior). By using video feedback, parents are enabled to reflect on their own behavior and the responses it triggers from their child. The VIPP‐SD is a relatively brief intervention program consisting of six home visits. A meta‐analysis including 12 randomized controlled trials showed that the VIPP‐SD is effective in enhancing parental sensitivity and sensitive discipline (combined effect size of *d* = 0.47), with smaller effects on child outcomes like attachment security and externalizing problem behavior in at‐risk samples (Juffer et al., 2017).

### 1.2 Parental sensitivity and face processing

Complex human behavior, such as parenting, results from an interplay of neural, cognitive, and emotional processes (see Pereira & Ferreira, 2016 for a review). As faces and facial expressions reveal information about the mental and emotional state as well as intentions of others, face processing capacities facilitate successful social interaction (Adolphs, 2003; Grady & Keightley, 2002; Zebrowitz, 2006). As perceiving, interpreting, and responding to children's emotional displays are at the core of both sensitivity and sensitive discipline (Ainsworth et al., 1974), neural processing of children's faces and facial expressions may be essential. Neural face processing can be examined noninvasively using electroencephalography (EEG), a method that records neural activity using electrodes placed on the scalp. Event‐related potentials (ERPs), that is, electrical activity in response to a specific event or stimulus (e.g., faces), can be used to quantify cognitive processing. Neural face processing is reflected in the N170, a negative‐going component of the ERP that peaks approximately 170 ms after stimulus onset and is thought to reflect the early stages of processing and encoding face configuration (Bentin et al., 1996; Botzel et al., 1994; 2016, 2016). Although more ERP components could be of interest, our preregistered focus is on neural face processing (Kolijn et al., 2017). In contrast with other ERP components, the N170 is face‐specific and thought to reflect the encoding/ processing of face configuration in the fusiform gyrus (see, e.g., Iidaka et al., 2006), which makes it the most appropriate ERP component in adherence to our registered study aim. In addition, the current study builds on our previous findings that showed an intervention effect on the N170 only, and not on the (not face‐specific) P1 and LPP (Kolijn et al., 2020).

The N170 in response to (emotional) infant and child faces varies between parents and nonparents with stronger N170 amplitudes in parents (Maupin et al., 2015; Proverbio et al., 2006; Weisman et al., 2012; Young et al., 2017). Differences have also been found depending on mothers' parenting capacities, with Child Protective Services (CPS) referred mothers not treated with an attachment‐based intervention (Bernard et al., 2015) and neglectful mothers (Rodrigo et al., 2011) failing to differentiate between facial expressions the way typical mothers, who show stronger neural responses for emotional over neutral faces, do. Interestingly, CPS‐referred mothers who received attachment‐based parenting support did show neural differentiation between children's emotional and neutral facial expression (Bernard et al., 2015). Moreover, stronger N170 amplitudes for emotional compared to neutral faces were associated with higher parental sensitivity, suggesting correspondence between neural differentiation for emotional infant cues and sensitive caregiving responses on a behavioral level.

### 1.3 Mediation: VIPP‐SD effects on maternal sensitivity via N170

We wanted to gain more insight in the mechanisms potentially mediating intervention effects on parenting behavior and, thus, parental characteristics that might explain the efficacy and success of such programs. In our randomized controlled study, we found more efficient face processing (reflected in smaller N170 amplitudes in response to children's emotional faces) in mothers who received the VIPP‐SD (Kolijn et al., 2020), suggesting that neural face processing is indeed affected by attachment‐based parenting support. Although direct associations between N170 amplitudes and maternal sensitivity have been reported (Bernard et al., 2015), no studies to date have—to the best of our knowledge—addressed the question whether face processing constitutes a mechanism for behavioral change in sensitivity. Therefore, the aim of the current study was to test whether changes in N170 amplitude mediate intervention effects on maternal sensitivity. Although the VIPP‐SD targets both parental sensitivity and sensitive discipline, we focus on intervention effects on maternal sensitivity in line with Bernard et al. (2015). We hypothesize that the intervention positively affects maternal sensitivity and that this change is mediated by a change in N170 amplitudes (i.e., the decreased amplitudes we reported on in Kolijn et al., 2020) over the course of the intervention.

## 2 METHOD

### 2.1 Participants

The Leiden Consortium on Individual Development (L‐CID) preschooler project is a longitudinal intervention study including families with 3‐ to 4‐year‐old twins (for details on the L‐CID design, see Euser et al., 2016). A random 40% was assigned to the intervention and 60% to a dummy intervention, the 40/60 ratio was chosen for feasibility reasons. The sensitive discipline feature of the VIPP‐SD targets the reduction and/or prevention of children's problem behavior. Although our sample was not selected for increased levels of problem behavior, parents of twins are challenged, as raising two same‐aged children at the same time increases child rearing demands (Klein, 2017; Lewin, 2016; Riva Crugnola et al., 2020). Therefore, parenting support is of particular relevance for these parents. The current study reports on a random subsample of mothers who were invited to participate in an additional part of the study focusing on the “parental brain” (see Kolijn et al., 2017, 2020). The current sample included 66 mothers (22 mothers in the intervention group and 44 mothers in the control group (due to the 40/60 randomization ratio in the larger L‐CID study, the groups differ in size) who were eligible and willing to participate in two additional EEG assessments. Sample characteristics are summarized in Table 1. Mothers were on average 37.29 years old (*SD* = 4.31), and their typically developing same‐sex twins were on average 4.66 years old (*SD* = 0.60, 52% girls) at the time of the current study's first assessment, that is, the pretest of maternal sensitivity. The majority of mothers were married or in a registered partnership (73%) or unmarried living together (23%), highly educated (77% had at least an undergraduate degree), and were born in the Netherlands (92%). Exclusion criteria for mothers were neurological and psychiatric diseases and use of psychoactive medication. Included mothers (*n = *66) did not differ from mothers who did not meet the inclusion criteria or declined to participate (*n* = 54) regarding background variables (i.e., marital status, maternal education, family SES, twin gender, and twin zygosity; all *p*s ≥ .10). Finally, the current study was registered (Kolijn et al., 2017) but it should be noted that our sample size deviates from the registered sample size (*n* = 100; 50 intervention group and 50 control group). Due to inclusion of an EEG pretest, we could only invite the families who were not randomized to either intervention or control group yet (*n* = 119) of which 66 participated in two additional EEG assessments (see Kolijn et al., 2020 for details). Consequently, the power to detect effects deviates from the registered protocol (see below, under “Statistical Analysis”).

**TABLE 1:** Sample characteristics, group differences/covariates

Sample characteristics	Total (*n* = 66)	Intervention (*n* = 22)	Control (*n* = 44)
	*M* (SD)	*M* (SD)	*M* (SD)
Age mother at T0	37.29 (4.31)	36.99 (4.23)	37.43 (4.38)
Age twin at T0	4.66 (0.60)	4.61 (0.66)	4.69 (0.57)
Age mother at T3	38.26 (4.30)	38.06 (4.25)	38.37 (4.38)
Age twin at T3	5.71 (0.60)	5.68 (0.64)	5.73 (0.58)
	%	%	%
Middle‐to‐high SES	92	95	91
Middle	38	36	39
High	55	59	52
Single parent	5	5	5
Twin girls	52	50	52
MZ twins	58	68	52
Group differences/covariates	*M* (SD)	*M* (SD)	*M* (SD)
BSI total mother	26.54 (5.20)	24.46 (4.29)	27.58 (5.34)*
Weeks between T0 and T1	33.14 (2.26)	32.13 (1.33)	33.65 (2.46)**
Weeks between T1 and Start	3.19 (1.71)	4.08 (1.76)	2.80 (1.55)*
Duration V/D	11.08 (2.89)	12.68 (4.20)	10.39 (1.74)*
Weeks between End V/D and T2	4.13 (3.51)	4.96 (5.34)	3.75 (2.20)
Weeks between T2 and T3	2.79 (1.97)	2.36 (1.44)	3.00 (2.17)

Note

Difference between intervention and control group: *p < .05, **p < .01, only significant covariates were added to the moderated mediation analyses in the sensitivity analyses (supplementary materials). “V/D” = VIPP‐SD or dummy intervention, “Start” = start of the intervention or dummy, “End” = End of the intervention or dummy.

### 2.2 Procedure

**FIGURE 1:**
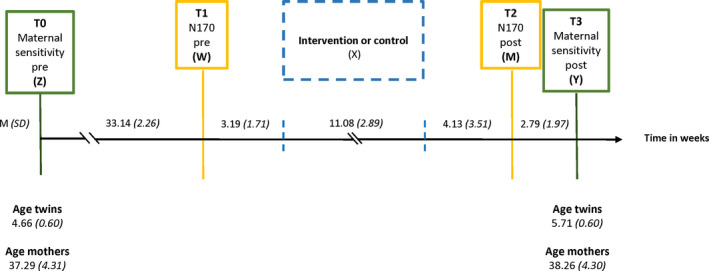
Time in weeks between assessments and intervention. M, W, X, Y, and Z refer to the variable abbreviations used in moderated mediation analysis (see Figure 2)

In the L‐CID project, families are followed for 6 years with yearly assessments, resulting in six waves existing of two pre‐ and four postintervention tests (Euser et al., 2016; Crone et al, 2020). In between wave two (i.e., second pretest) and three (i.e., the first posttest), families were randomized to receive either the VIPP‐SD program or a dummy intervention with six phone calls (see below). Approximately 2 weeks before and 2 weeks after the intervention, the current subsample participated in additional EEG assessments (see Figure 1 for an overview of the sequence of assessments and the intervention). The current study includes data from four L‐CID assessments: Wave 2 maternal sensitivity data (preintervention; first green box in Figure 1), maternal EEG data from the additional pre‐ and postintervention assessments (yellow boxes in Figure 1), and Wave 3 maternal sensitivity data (postintervention; second green box in Figure 1). All visits took place at the Leiden University Child and Family laboratory. At the start of the L‐CID study, participants signed informed consent. For the additional EEG assessments, mothers signed an additional informed consent form at the start of the first EEG visit. At the end of each visit, participants received a financial reimbursement (€50 for each yearly visit and small presents for the children and €20 for each EEG visit) and their travel expenses were compensated. The Institutional Review Board of Leiden University's Institute of Education and Child Studies and the Central Committee on Research Involving Human Subjects in the Netherlands (CCMO; NL49069.000.14) approved all assessments.

### 2.3 Intervention program

The VIPP‐SD (Juffer et al., 2017) is based on Bowlby's attachment theory (Bowlby, 1982, 1988) and Patterson's social learning theory (1982), and includes sensitivity and sensitive discipline themes (see Juffer, Bakermans‐Kranenburg, & Van IJzendoorn,^2008^ for an overview). The intervention consists of one start‐up home visit followed by five biweekly home visits during which parent–child interactions are videotaped and positive feedback is provided. In between sessions, the intervener reviews the videos and, guided by positive parent–child interaction episodes, prepares the feedback. During every subsequent visit, the mother and intervener reflect on the positive episodes of the previous visit. The use of video feedback is a powerful element of the intervention as mothers can identify with the material, thereby using mother's own as well as their children's behavior as a model for behavioral change. In addition, an empathic intervener–parent relationship and an emphasis on mothers being the expert on their own children are pivotal elements of the intervention. The implementation of the VIPP‐SD in the current study required sample‐specific adjustments, and accordingly, the VIPP‐SD manual was adapted for families with twins (see Euser et al., 2016 for details). All interveners were extensively trained by certified VIPP trainers in using the VIPP‐SD version 3.0 manual (Juffer, Bakermans‐Kranenburg, & Van IJzendoorn, 2008) and running home visits with twins. On average, mothers who participated in the VIPP‐SD program completed 5.63 visits (*SD* = 0.96, *n* = 19; three participants did not start the intervention).

### 2.4 Control condition

To control for the potential effect of receiving expert attention, participants randomized to the control condition were contacted by phone six times. Following a standardized protocol, trained research assistants asked parents to report on their twins' general development by using a semi‐structured interview. On average, the control group completed 5.89 phone calls (*SD* = 0.32, *n* = 44), which was not different from the number of visits completed by the intervention group (*p* = .27).

### 2.5 Measures

#### 2.5.1 Maternal sensitivity

To observe maternal sensitivity, parent–child dyads performed a computerized version of the Etch‐a‐Sketch task (Deater‐Deckard, 2000; Stevenson‐Hinde & Shouldice, 1995; Vrijhof et al., 2019) in which they were instructed to digitally replicate three printed drawings (ascending in difficulty). The task was performed on a laptop, using four buttons: One button pair controlled the lines going up and down, the second button pair controlled the line going left or right. Before starting the task, parent and child decided among themselves who would control which pair of buttons. This task is particularly suited to elicit parent–child interaction, as parent and child need to cooperate actively to succeed at the task. The dyad started with replicating the first drawing, and after three minutes, an audio cue signaled parent and child to start replicating the second drawing if they had not finished drawing the first. We videotaped the interaction and recorded the actions on the laptop screen. Afterward, the two recordings were integrated side by side into a single video.

The revised Erickson 7‐point rating scales for supportive presence (1 = parent completely fails to be supportive to the child, 7 = parent skillfully provides support throughout the session) and intrusiveness (1 = parent allows the child sufficient time to explore and to solve things on their own, 7 = parent is highly intrusive; her/his agenda clearly has precedence over the child's wishes; Egeland et al., 1990) were used to rate maternal sensitivity. In total, eight coders—trained by an expert coder—were involved in coding the video's (see Table 2 for intercoder reliability). Videos of co‐twins and videos from the same family in different assessments were never coded by the same coder.

**TABLE 2:** Values of the ICC reliability sets

	ICC with expert coder *M* (range)	ICC among the 8 coders *M* (range)
Pretest		
Supportive presence	0.83 (0.76–0.89)	0.83 (0.76–0.89)
Intrusiveness	0.77 (0.72–0.81)	0.79 (0.72–0.87)
Posttest		
Supportive presence	0.74 (0.68–0.77)	0.71 (0.64–0.78)
Intrusiveness	0.75 (0.68–0.80)	0.76 (0.61–0.84)

Note

Number of videos in reliability set pretest = 40 and in reliability set posttest = 48.

For interpretation purposes, the intrusiveness scale was reversed into nonintrusiveness so that higher scores represented higher levels of maternal sensitivity on both scales. As the current study involved families with twins, every mother received four scores per maternal sensitivity assessment (i.e., two scales × two children) that were used to compute one maternal sensitivity score per mother, per assessment using the following procedure. Supportive presence and nonintrusiveness scores within each child were significantly related (*r* = .55 and .54, *p* = <.01 for the pretest and *r* = .58, *p* = .01 and .34, *p* < .01 for the posttest). Therefore, we first created one average maternal sensitivity score per co‐twin per assessment. These two scores were significantly related within mothers (*r* = .39, *p* = <.01 for the pretest and *r* = .51, *p* = <.01 for the posttest). Consequently, one overall maternal sensitivity score per assessment was computed, resulting in one pre‐ and one posttest maternal sensitivity score. On average, mothers scored 4.12 (*SD = *1.09) at the pretest and 4.39 (*SD* = 1.09) at the posttest. The data were approximately normally distributed (|skewness| < 1, |kurtosis| < 1), and no outliers were present (no *z*‐scores > 3.29 or <−3.29).

### 2.6 EEG paradigm stimuli

Stimuli were obtained from the Child Affective Facial Expression set (CAFE; LoBue, 2014) that contains full‐color photographs of young children (face only) expressing a variety of emotional facial expressions. The children in the CAFE set (age 2–8 years) are approximately the same age as the children of the participants in our study. Because the majority of children in our sample were Caucasian, we selected the “White” subset of the CAFE set. To avoid confounding facial expression with child identity, we initially selected only pictures of children for whom the facial expressions neutral, happy, and angry were reported as valid by LoBue and Thrasher (2015), which was the case for pictures of 22 children (10 girls and 12 boys). In contrast with prior studies, we included anger instead of sadness due to the VIPP‐SD's focus on responding to children's noncompliant, challenging behavior that often involves anger but usually not sadness (see also Kolijn et al., 2020). We matched the selected photographs on size and luminosity and after a convenience sample of 16 faculty members of Leiden University rated the pictures on emotion (for details, see Kolijn et al., 2020), we included photographs of 16 children (nine boys, seven girls) in our final stimulus set.

### 2.7 EEG face processing paradigm

In total, 144 pictures (i.e., 16 children × three facial expressions × three presentations) were presented to the participants in a quasi‐random order, with the restriction that the same emotion could not occur more than four times in a row. Stimuli were presented on a black background on a computer monitor in a dimly lit and sound attenuated room. A white fixation cross on a black screen started every trial (duration varied randomly between 800 and 1,200 ms) after which a picture (6.60 × 8.10° visual angle) was presented for 1,000 ms. Participants were offered a 10‐s break to rest their eyes after every 24th trial. To maintain participants' attention, participants were asked once during every block of 24 trials (varying randomly between the 5th and 24th trial) to indicate the gender of the child in the picture by a button press. The majority (86%) of the sample answered all gender questions correctly (the remaining 14% answered one gender question incorrectly), and accuracy did not differ between the intervention (*M* = 5.86 correct answers, *SD* = 0.35) and control group (*M* = 5.86 correct answers, *SD* = 0.35). Participants were instructed not to move and to look straight at the screen. The paradigm took about 8 min to complete.

### 2.8 ERPs

While participants viewed the pictures, their EEG was recorded using NetStation software (RRID: SCR_002453) and 129‐channel Hydrocel Geodesic Sensor Nets (Electrical Geodesics, Inc.). The signal was amplified using a NetAmps300 amplifier, low‐pass filtered at 200 Hz, and digitized at a rate of 500 Hz. Cz was used as the reference during recording. Impedances were kept below 50 kΩ. A 0.3 Hz high‐pass filter (99.9% pass‐band gain, 0.1% stop‐band gain, 1.5 Hz roll‐off) was applied before data were exported to be processed further using Brain Vision Analyzer 2.0 software (Brain Products GmbH). A 30 Hz low‐pass filter (−3 dB, 48dB/octave) was applied, and data were rereferenced to the average of activity in all 129 channels. The EEG data in the current study overlap with the data in Kolijn et al. (2020) and were analyzed in the same way.

In short, 1,200 ms segments extending from 200 ms before to 1,000 ms after stimulus onset were extracted and corrected for ocular artifacts using independent component analysis (ICA). Segments containing residual artifacts were removed if the difference between the maximum and minimum activity in the left (el. 25–el. 127) and right (el. 8–el. 126) eye channels was larger than 100 μV within any 200 ms window or if activity in the horizontal eye channel (el. 125–el. 128) was larger than 60 μV within any 200‐ms window. When the difference between the minimum and maximum activity was larger than 150 μV in a particular channel during a particular segment, we removed that channel from that segment. Finally, an average ERP waveform was created for every emotion (i.e., neutral, happy, and angry) for each assessment (pre‐ and posttest). For the pretest (*n* = 66), participants had on average 45 (*SD* = 5.74, range: 23–48), 44 (*SD* = 5.86, range: 21–48), and 45 (*SD = *5.39 range: 23–48) artifact‐free trials in response to neutral, happy, and angry stimuli, respectively, without significant differences between the intervention and control group (all *t*_s_ ≤ 1.64, all *p*_s_ ≥ .11). For the posttest (*n* = 60; five persons did not participate in the posttest and one participants' session was aborted, see “statistical analyses”), these numbers were 44 (*SD* = 7.52, range: 6–48), 44 (*SD* = 7.88, range 9–48) and 44 (*SD* = 7.30, range 7–48), again without differences between the intervention and control group (all *t*_s_ ≤ 1.46, all *p*_s_ ≥ .15).

Based on a priori considerations and inspection of grandaverage waveforms (i.e., the ERP averaged across groups, conditions and sessions; see Kolijn et al., 2020 for a detailed description), we quantified the N170 as the average voltage within the 138–168 ms time window across electrode sites 58, 64, and 65 (left N170), and 90, 95 and 96 (right N170). After winsorizing two outliers (*z* = 3.75 for posttest left happy, *z* = –3.63 for posttest left angry; Tab achnick & Fidell, 2013), the data were approximately normally distributed (|skewness| < 1, |kurtosis| < 2) without outliers (no *z*‐scores > 3.29 or <–3.29). As reported in Kolijn et al. (2020), the intervention affected N170 amplitudes regardless of facial expression or hemisphere, and therefore, we created one overall pre‐ and one overall postintervention measure of N170 amplitude by averaging across the three facial expressions and both hemispheres. These two variables were approximately normally distributed (|skewness| < 1, |kurtosis| < 1) without outliers (no *z*‐scores > 3.29 or <−3.29).

### 2.9 Covariates

There were four potential confounders, which we included as covariates in the sensitivity analyses: (a) time in weeks between T0 and T1, (b) time in weeks between T1 and start of the VIPP‐SD program or dummy intervention, (c) the duration of the VIPP‐SD program or dummy intervention, and (d) level of self‐reported psychopathological symptoms measured with the Brief Symptom Inventory (BSI; Derogatis, 1975; De Beurs & Zitman 2005).

### 2.10 Statistical analyses

A total of seven mothers (three in the intervention group and four in the control group) had missing EEG posttest data: Five did not participate, one participants' session was aborted due to illness, and one participant did not provide sufficient artifact‐free data (i.e., at least 10 artifact‐free trials per condition). For maternal sensitivity, two mothers had missing data on the posttest (both in the control group). Missing data were handled by carrying the last observation forward (i.e., pretest, see Little & Yau., 1996) to ensure a complete dataset across all assessments. Three participants did not start the intervention due to time constraints or personal circumstances, but conform the ITT approach we analyzed them in the group to which they were randomly assigned. Finally, after finishing data collection, one participant (control group) reported use of psychoactive medication, but she was included in the analyses conform the intent to treat (ITT) approach (Kolijn et al., 2020).

**FIGURE 2:**
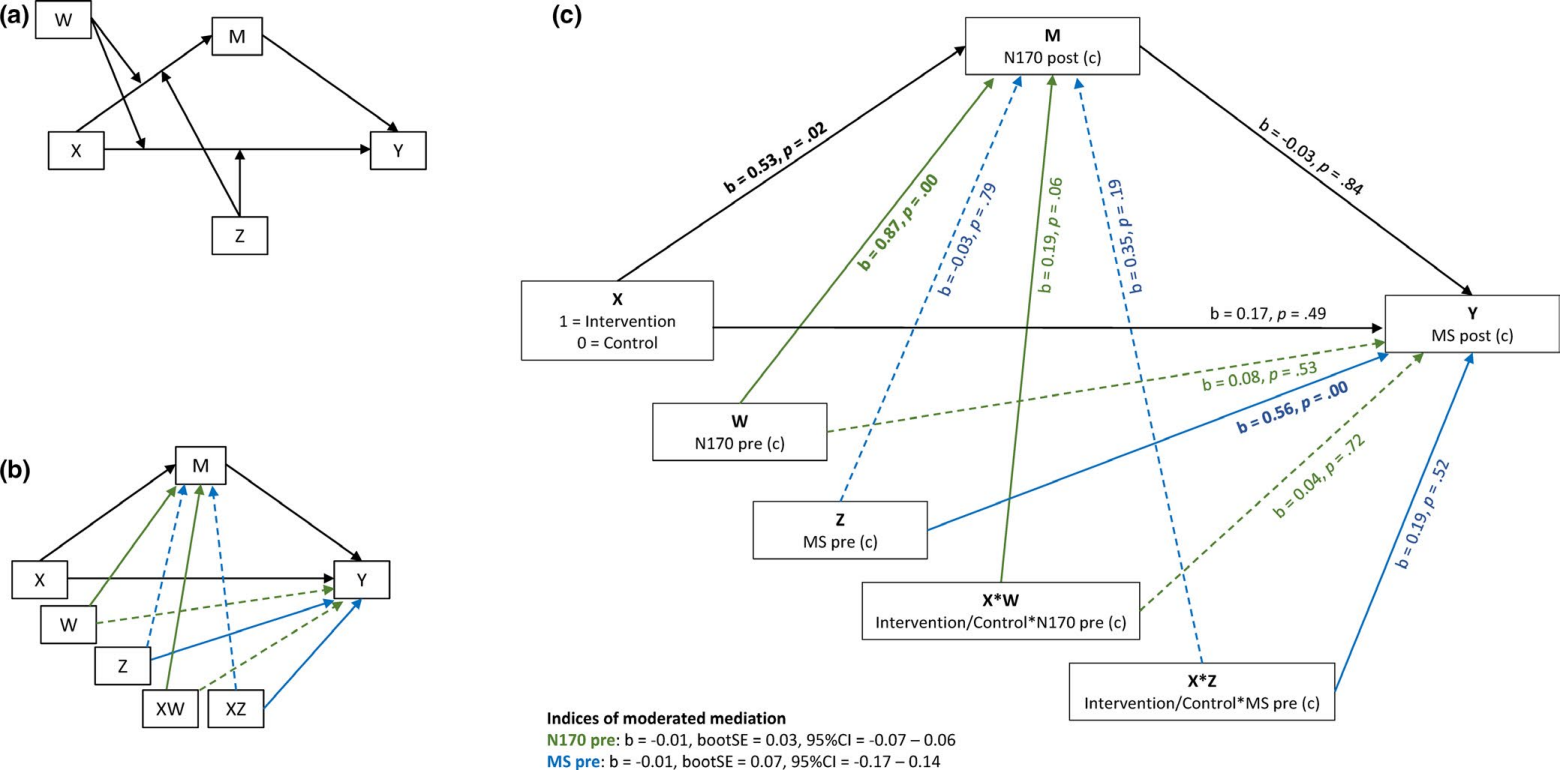
Moderated mediation as modeled in PROCESS model 10. Panel a: conceptual model, panel b: statistical model, with green arrows representing the (interaction) effects of the pretest of N170 amplitudes and blue arrows representing effects of pretest of maternal sensitivity. The solid lines in Panel b represent the associations that are most relevant to our research question. Panel b's corresponding variable names, interaction terms, and regression weights can be found in panel c. Panel c: Results of the moderated mediation analysis (PROCESS model 10) with the most relevant associations represented by the solid lines. Significant associations are indicated in bold font, (c) = centered

We performed two sets of analyses to answer our research question. First, we performed a first‐stage moderated mediation analysis using model 10 in Hayes' PROCESS macro (Hayes, 2018; see Figure 2 panel A for the conceptual model and panel B for the statistical model). As illustrated in Figure 2, PROCESS' model 10 performs a series of regression analyses testing whether a difference between the intervention and control group (X) on maternal sensitivity at posttest (Y) is mediated by N170 amplitude at posttest (M) while taking pretest N170 amplitudes (W) and pretest maternal sensitivity (Z) into account. More specifically, the indices of partial moderated mediation indicate whether one moderator (N170 amplitude [W] or sensitivity [Z]) is related to the size of the indirect effect (i.e., effect of the intervention on posttest maternal sensitivity through posttest N170 amplitudes), independent of the other moderator (Hayes, 2018). An intervention effect on N170 amplitude (the mediator) can appear as a significant interaction term X × W and/or a significant main effect of experimental condition. Similarly, an intervention effect on maternal sensitivity (the outcome) can manifest itself as a significant interaction term X × Z and/or a significant main effect of experimental condition. We coded the between‐subjects factor as 0 for the control group and 1 for the intervention group. Moderated mediation effects were tested using the percentile bootstrap method with 10,000 runs, and we centered the continuous variables before the analysis by subtracting the group mean from every individual score. Testing the total effect of the intervention on maternal sensitivity with *α* = .05 in a sample of 66 mothers, the power to detect a medium‐sized effect is 0.51 (G*Power 3.1.9.2; Faul et al., 2009). The power to detect substantial mediation is at least similar, and often larger than the power to detect the overall effect (Kenny & Judd, 2014).

The moderated mediation analysis approaches our association of interest most closely, but not to the full extent, as the analysis fails to take into account the moderating effects of both moderators (i.e., pretest N170 and pretest sensitivity) at the same time. Moreover, PROCESS' model 10 estimates and tests many coefficients that are not relevant for our research question (dotted lines Figure 2), affecting the power of the analysis, which is especially relevant given our relatively small sample. Therefore, we performed additional two‐condition mediation analyses per group using Montoya and Hayes' (2017) MEMORE macro for SPSS. The MEMORE model does not include a between‐subjects (intervention vs. control group) comparison. Thus, these analyses tested whether, within the intervention and control groups separately, a change in the dependent variable maternal sensitivity from pre‐ to posttest is mediated by a pre‐ to posttest change in N170 amplitude. We tested the indirect effect by using the percentile bootstrap method with 10,000 runs, and we used noncentered variables (essential for these analyses; see Montoya & Hayes, 2017). Alpha was set to .05 in all analyses.

In order to test the robustness of associations, we performed sensitivity analyses by repeating the moderated mediation analysis in which we included the variables on which the intervention and control group significantly differed as covariates. The outcomes of these analyses are presented in the appendix, and the conclusions are in line with our main findings.

## 3 RESULTS

### 3.1 Moderated mediation analysis

**FIGURE 3:**
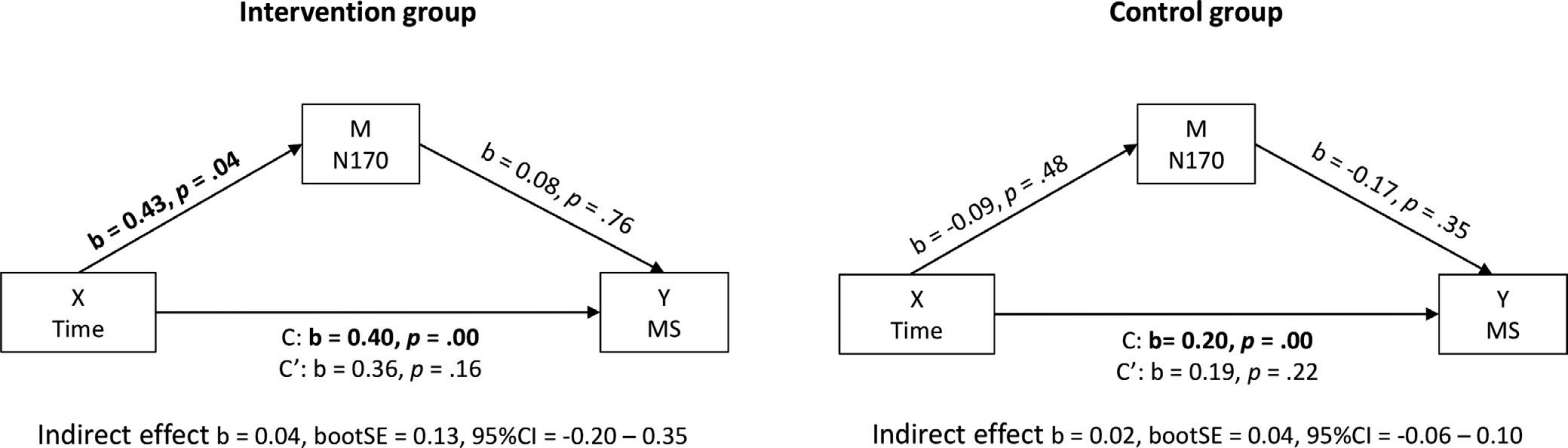
Results of the two‐condition mediator analysis (MEMORE) per group. Significant associations are displayed in bold. The mediator represents the N170 difference score, and the outcome measure represents the maternal sensitivity difference score (i.e., post‐ vs. pretest)

Descriptive statistics of all variables can be found in Table 3. We used Hayes' PROCESS macro model 10 for moderated mediation analysis to test whether post‐N170 amplitudes mediated the effect of the VIPP‐SD on postmaternal sensitivity. Results are illustrated in Figure 3. Both pre‐ and posttest N170 amplitudes *r* = .87, *p* < .01 and pre‐ and posttest maternal sensitivity *r* = .56, *p* < .01 were significantly related. There was no intervention effect on maternal sensitivity, as indicated by the absence of a main effect of condition (*b* = 0.17, *p* = .49) on posttest maternal sensitivity and the absence of an interaction effect of group and pretest maternal sensitivity (*b* = 0.19, *p* = .52 [X × Z, illustrated in blue in Figure 2 panel C]). The intervention and control groups were significantly different on N170 amplitudes at posttest (*b* = 0.53, *p* = .02; smaller N170 amplitudes in the intervention than the control group). The interaction effect of condition and pretest N170 (X × W, illustrated in green in Figure 2 panel C) on posttest N170 amplitudes was marginal (*b* = 0.19, *p* = .06). Furthermore, we did not find a significant association between the N170 at posttest and maternal sensitivity at posttest (*b* = −0.03, *p* = .84). Moreover, the indices of partial moderated mediation provided no evidence for our hypothesis, as these were not significant: Independent of pretest maternal sensitivity, the indirect effect of the intervention on posttest maternal sensitivity through posttest N170 amplitudes was not significantly moderated by the pretest N170 amplitudes (*b* = −0.01, bootstrapped *SE* = 0.03, 95% confidence interval (CI): −0.07 to 0.06). Independent of pretest N170 amplitudes, the indirect effect of the intervention on posttest maternal sensitivity through posttest N170 amplitudes was not significantly moderated by the pretest maternal sensitivity (*b* = −0.01, bootstrapped *SE* = 0.07, 95% (CI): −0.17 to 0.14).

**TABLE 3:** Descriptive statistics of maternal sensitivity and N170 amplitudes

Variable	Total (*n* = 66)	Intervention (*n* = 22)	Control (*n* = 44)
	*M* (SD)	*M* (SD)	*M* (SD)
Pretest			
Sensitivity	4.12 (1.09)	4.09 (0.79)	4.14 (1.22)
N170	−0.38 (2.36)	−0.23 (2.49)	−0.45 (2.31)
Posttest			
Sensitivity	4.39 (1.09)	4.49 (1.15)	4.34 (1.08)
N170	−0.29 (2.39)	0.20 (2.75)	−0.54 (2.17)

### 3.2 Two‐condition mediator analyses

Using Montoya & Hayes' MEMORE macro, we performed mediation analyses for the intervention and control group separately. The results are illustrated in Figure 3. In the intervention group, we found a significant effect of time on maternal sensitivity (total effect: *b* = 0.40, *SE* = 0.05, *p* < .01) with significantly higher maternal sensitivity scores at the posttest compared to the pretest. We also found a significant effect of time on N170 amplitude (*b* = 0.43, *p* = .04), with smaller (i.e., less negative) N170 amplitudes at the posttest compared to the pretest. There was no significant association between the pre‐ to posttest changes in N170 amplitude and maternal sensitivity (*b* = 0.08, *p* = .76). Although the direct effect of time, taking the N170 into account, was not significant (*b* = 0.36, *SE* = 0.25, *p* = .16), we did not find evidence for mediation as the indirect effect was not significant either, *b* = 0.04, bootstrapped *SE* = 0.13, 95% CI: −0.20 to 0.35. We also found an effect of time on maternal sensitivity in the control group (total effect: *b* = 0.20, *SE* = 0.02, *p* < .01) with higher maternal sensitivity scores at the posttest compared to the pretest. There was no effect of time on the N170 (*b* = −0.09, *p* = .48) or an association between the difference in N170 amplitude between pre‐ and posttest and the difference in maternal sensitivity from pre‐ to posttest (*b* = −0.17, *p* = .35). We did not find evidence for mediation as the indirect effect was not significant (*b* = 0.02, bootstrapped *SE* = 0.04, 95% CI: −0.06 to 0.10), even though the direct effect was not significant either (*b* = 0.19, *SE* = 0.15, *p* = .22).

## 4 DISCUSSION

In previous RCTs, the VIPP‐SD has been found to be effective in enhancing parental sensitivity. Our aim was to gain insight into neural processes that potentially contribute to the behavioral effects. We hypothesized that the intervention would promote maternal sensitivity by enhancing mothers' ability to accurately perceive and interpret their children's (emotional) needs, based on their children's facial expressions that reveal important nonverbal information about mental states (Zebrowitz, 2006). By “training” mothers' ability to read or scan their children's faces for emotional needs and automating cognitive processing, the intervention may result in less effortful, more efficient neural face processing, as we observed previously (Kolijn et al., 2020). Following up on that finding, we conducted mediation analyses to test whether such gains in neural face processing enhanced maternal sensitivity to their children's signals. However, in the current subsample of the larger L‐CID sample the VIPP‐SD did not significantly enhance maternal sensitivity, and neural face processing was not involved as a mediator. Nonetheless, in accordance with our previously reported finding of a VIPP‐SD effect on the N170 (Kolijn et al., 2020) the two‐condition mediation analyses demonstrated that the time effect on N170 amplitudes (smaller at posttest) was present in the intervention group but absent in the control group. The more sophisticated moderated mediation analysis showed that N170 amplitudes at posttest were smaller in the intervention compared to the control group while controlling for pretest N170 amplitudes. To date, only a couple of studies have investigated neurobiological factors that may be involved in intervention effects on parenting (Bernard et al., 2015; Swain et al., 2014, 2017), highlighting the current lack of understanding of these processes. The current EEG study is the first—to the best of our knowledge—to employ a strong study design, using a randomized controlled trial including pre‐ and postintervention measures, and including mothers of twins to enhance the reliability of the behavioral assessments.

One explanation for the absence of a mediation effect may lie in the fact that the time window between the EEG posttest and the maternal sensitivity posttest was rather short, less than 3 weeks on average. It may well be that it takes a longer period of time for effects on neural face processing to result in observable changes in complex behavior. However, it is also possible that our results reflect true null effects. Assuming a “true” null result, our study showed notable differences with studies that reported intervention effects on both neural and behavioral measures. Thus, the current study does not constitute an exact but rather a varied replication (Van IJzendoorn, 1994) of the previous studies that were the basis for the formulation of our mediation hypothesis. The most notable differences involve sample characteristics. Whereas our study included a sample of advantaged families (as indicated by the high proportion of two‐parent families and a predominantly middle to high educational level), previous research studied high‐risk and/or disadvantaged samples (Swain et al., 2014, 2017; Kim, Capistrano, Erhart, Gray‐Schiff, & Xu, 2017) characterized by, for example, neglect and/or maltreatment (Bernard et al., 2015) or substance use (Suchman et al., 2008). Such differences may help explain the absence of behavioral intervention effects in the current study, but may also be relevant for the neurocognitive effects that can be observed. As evidenced by our previous results and corroborated by our current findings, our intervention led to a reduction of neural effort required to process children's faces and smaller N170 amplitudes (Kolijn et al., 2020), whereas enhanced N170 (and LPP) amplitudes and reflecting increased neural processing of infant faces, was observed in high‐risk samples (N170 and LPP; Bernard et al., 2015). Whether face processing is differentially impacted in high‐risk mothers, leading to different neurocognitive outcomes and (resulting) behavioral effects of intervention programs depending on the population involved, is an outstanding issue and constitutes an important topic for future research. Furthermore, other differences between our own findings and those of Bernard et al. deserve attention here. First, whereas stronger (i.e., more negative) N170 amplitudes were found for emotional over neutral faces after the ABC intervention (Bernard et al., 2015), the N170 in our study was unaffected by emotional expression (see Kolijn et al., 2020). Regardless of intervention effects, many (Hinojosa, Mercado & Carretié, 2015) but not all (Noll, Mayes, & Rutherford, 2012; Malak, Crowley, Mayes and Rutherford, 2015; Rutherford, Maupin, Landi, Potenza, & Mayes, 2017) studies have found the N170 to be affected by emotional expressions, in particular their intensity. To what extent emotional expressions play a role remains important to address in future research. Second, variation in task design possibly played a role here as well. Whereas Bernard et al. (2015) used a categorization task in which mothers viewed children's faces and subsequently classified the emotion expressed, the mothers in our study passively viewed the pictures and there was no “task” involved. Task demands, especially in the degree to which faces and/or facial expressions are relevant for the task's demands, might affect N170 effects (see also Kolijn et al., 2020; Huffmeijer et al., 2018), and this may have contributed to the divergent findings.

Although our sample was not high risk, it did consist of families with twins. Parents of twins experience increased parenting demands, since two same‐aged children claim their attention. Importantly, research in twin families has revealed higher levels of (parenting) stress, depression, exhaustion, and perceived parenting difficulty (Andrade et al., 2014; Damato, 2005; Lutz et al., 2012; Olivenness et al., 2005). Additionally, parents face parenting challenges resulting from twin interrelationships—affecting developmental trajectories of identity formation and forming (peer) relationships (Klein, 2017; Lewin, 2016). Nevertheless, our sample was characterized by buffering factors such as the virtual absence of single parenthood (95%), middle‐to‐high SES, and low parental psychopathology, pointing to substantial differences with high‐risk samples.

An important strength of our study is the RCT design with pretests and posttests. The inclusion of a pretest is essential to examine any manipulation‐induced change over time. Besides quantification of baseline levels, pretests in RCTs can reveal existing preintervention differences—even after random assignment—that may confound differences in posttest measures. This is particularly relevant when samples of a modest size are used, as chance factors might lead to substantial pretest differences. Still, researchers should be aware of the paradox that pretests are included to secure internal validity of the design, but at the same time pose inherent threats to other aspects of both internal and external validity (Hartley, 1973; Hoogstraten, 1979; Kim & Willson, 2010). Participating in a pretest gives away what is being measured at posttest by “pretest sensitization” (i.e., the potential or actual preintervention assessment effect on participants performance; Willson & Kim, 2010; Kim & Willson, 2010; see Song & Ward, 2015 for a review). In our study, the pretest may have reduced variation between the intervention and control group as a result of similar pretest sensitization. The pretest could have primed the intervention targets and differentially (de)motivated parents (Rahmqvist et al., 2014), whereas not including a pretest would have kept all parents uninformed. The Solomon four‐group design (Solomon, 1949) in which participants are randomly assigned to one of four groups with and without pretest enables researchers to compare all possibilities of pre‐ and/or posttest in combination with or without intervention and examine effects of including a pretest. However, a Solomon four‐group design suffers from statistical issues (Sawiloskwy et al., 1994) and has limited feasibility (Michel & Haight, 1996).

### 4.1 Considerations for future research

Null findings deserve more attention in the scientific literature, as not publishing null results might lead to substantial publication bias and to a waste of scarce research resources (Ferguson & Heene, 2012). Fortunately, the importance of null findings and failed replications has become increasingly recognized over the past years (Ferguson & Heene, 2012; Landis et al., 2014; Mehler et al., 2019). The current study was registered (Kolijn et al., 2017), making a priori choices about the methodology and analytic strategies explicit, limiting the chance of selective use of post hoc analytic strategies that favor significance, and preventing selective publication of significant outcomes. Preregistration is a promising tool in decreasing publication bias, thereby contributing to the reproducibility and credibility of research efforts (Van 't Veer & Giner‐Sorolla, 2016). Indeed, registered studies show increased rates of published null findings (Allen & Mehler, 2018; Kaplan & Irvin, 2015). With respect to publication of the current null findings, the absence of mediation may promote the idea that the intervention may operate on other levels than we tested. This generates hypotheses and recommendations for future research. For instance, the VIPP‐SD themes “speaking for the child” and “sharing emotions” could increase cognitive processes that are involved in perspective taking/mentalizing and internal verbalization that in turn could promote maternal sensitivity on a behavioral level.

Future research may also attend to some limitations of the current study. Whereas our sample size was sufficient to detect neural effects (cf. Huffmeijer et al., 2014), it may have been too small to detect the effects on a behavioral level (Tab orsky, 2010). However, the power to detect substantial mediation is often larger than the power for direct effects (Kenny & Judd, 2014) and sample size problems are limited in within‐subjects designs (Kenny & Judd, 2019; Thompson & Campbell, 2004; Van IJzendoorn & Bakermans‐Kranenburg, 2016). Another point relates to the “task” that we used in our EEG paradigm, passive viewing of children's faces. Most previous studies focused on neural processing in mothers of infants and included stimuli depicting full‐blown emotional expressions or infant cry sounds (Maupin et al., 2015). These represent signals that are highly relevant for parents of infants who have no language as a means to express their emotions. Our study included mothers of preschoolers who can express their needs in ways beyond purely nonverbal means. We included age‐appropriate stimuli with high ecological validity for parents with preschoolers, but these were not accompanied or followed by verbal comments. Adding verbal expressions could be considered in future research.

### 4.2 Conclusion

Overall, we conclude that the effect of the parenting support program VIPP‐SD on neural activity was not accompanied by an intervention effect on parenting sensitivity in the current sample of families with twin preschoolers. The time required for neural changes to result in observable changes in complex behavior may be longer than the time window of the current study, or behavioral change may be more subtle than can be detected using a relatively small sample. Although our findings do not converge with those of previous studies, our uniquely strong study design strengthens our belief that our (null) findings will enrich the current scientific debate concerning mediation of behavioral intervention effects by changes in neurocognitive processes. At the very least, they emphasize the complexity of bridging the brain‐behavior gap in randomized interventions. Ultimately, publishing null findings will contribute substantially to scientific knowledge and stimulate scientific practice in moving toward replicable science, based on registered studies and open for outcomes that fail to take the magic hurdle of *p* < .05.

## CONFLICT OF INTEREST

The authors report no conflicts of interests.

## AUTHOR CONTRIBUTION

Laura Kolijn collected and analyzed the data and drafted the manuscript. Bianca van den Bulk contributed to the development of the tasks and to the study design. Saskia Euser contributed to the study design and coordinated coding activities of maternal sensitivity. Marian Bakermans‐Kranenburg and Marinus van IJzendoorn conceived the study and contributed to the study design. Rens Huffmeijer contributed to the development of the task, the study design, and data analyses. All authors contributed to revising and refining the manuscript and read and approved the final manuscript.

### PEER REVIEW

The peer review history for this article is available at https://publons.com/publon/10.1002/brb3.1972.

## Supporting information

Supplementary Material

## Data Availability

The data that support the findings of this study are available from the corresponding author upon reasonable request. All experiments reported here were preregistered.
