# Supplementary material for: Does neural face processing explain effects of an attachment‐based intervention on maternal sensitivity? A randomized controlled study including pre‐ and postintervention measures
Source: Brain Behav. 2021 Dec 8;12(1):e01972. doi: 10.1002/brb3.1972 (PMC8785642; doi:10.1002/brb3.1972)
Supplement: Supplementary file 1 — Supplementary Material [file BRB3-12-e01972-s001.pdf]

Supplementary Materials

Sensitivity analyses

Moderated mediation analysis including four covariates described under 'Statistical Analyses'.

Hayes' PROCESS macro model 10 was used for moderated mediation analysis to test whether post-N170 amplitudes mediated the effect of the VIPP-SD on post-maternal sensitivity while including four covariates. Both pre- and post-test N170 amplitudes $r = .87$, $p < .01$ and pre- and post-test maternal sensitivity $r = .56$, $p < .01$ were significantly related. On maternal sensitivity at post-test, the intervention and control groups were not significantly different (b = 0.01, $p = .98$). The interaction effect of group and pre-test maternal sensitivity (the actual intervention effect [X*Z]) on post-test maternal sensitivity was not significant (b = 0.02, $p = .94$), thus the change in maternal sensitivity from pre-test to post-test did not differ between the intervention and control group. On N170 amplitudes at post-test, the intervention and control groups were not significantly different (b = 0.31, $p = .32$), neither was the interaction effect of condition and pre-test N170 (the intervention effect [X*W]) on post-test N170 amplitudes (b = 0.20, $p = .08$). Furthermore, we did not find a significant association between the N170 at post-test and maternal sensitivity at post-test (b = -.07, $p = .62$). Moreover, the indices of partial moderated mediation provided no evidence for our hypothesis, as these were not significant: Independent of pre-test maternal sensitivity, the indirect effect of the intervention on post-test maternal sensitivity through post-test N170 amplitudes was not significantly moderated by the pre-test N170 amplitudes (b = -0.01, bootstrapped SE = 0.04, 95% confidence interval (CI): -0.11 - 0.06). Independent of pre-test N170 amplitudes, the indirect effect of the intervention on post-test maternal sensitivity

through post-test N170 amplitudes was not significantly moderated by the pre-test maternal sensitivity (b = -0.02, bootstrapped SE = 0.09, 95% (CI): -0.26 – 0.13).

Furthermore, all covariates were unrelated to post-test N170 amplitudes at post-test (BSI: b = -0.00, *p* = .85; Time between T0 and T1: b = -0.01, *p* = .93; Time between T1 and start of the condition: b = 0.08, *p* = .39; Duration of the condition: b = 0.04, *p* = .38) and post-test maternal sensitivity (BSI: b = -0.04, *p* = .14; Time between T0 and T1: b = -0.06, *p* = .34; Time between T1 and start of the condition: b = 0.03, *p* = .73; Duration of the condition: b = 0.03, *p* = .58).
